# Body in memory: the role of bodily self-consciousness in episodic memory and autonoetic consciousness

**DOI:** 10.1098/rstb.2025.0167

**Published:** 2026-11-10

**Authors:** Qiaoyue Ren, Andreea M. Gui-Bordeianu, Olaf Blanke

**Affiliations:** ^1^ Laboratory of Cognitive Neuroscience (LNCO), Neuro-X Institute and Brain Mind Institute, École Polytechnique Fédérale de Lausanne (EPFL), Lausanne, Switzerland; ^2^ Department of Clinical Neurosciences, Geneva University Hospital, Geneva, Switzerland

**Keywords:** bodily self-consciousness, episodic memory, autonoetic consciousness, sense of self

## Abstract

Episodic memory (EM) enables the conscious recollection of personally experienced events, including contextual features such as visual, auditory and emotional details. However, the role of bodily signals has been largely neglected in EM, although the observer's body provides a rich bodily context during the encoding, storage and recall of events, and has recently been associated with a bodily form of self-consciousness (bodily self-consciousness; BSC). Here, we introduce key concepts and findings on BSC and then summarize recent experimental paradigms, as well as behavioural and neural evidence, on how BSC impacts EM. This includes autonoetic consciousness, a core EM feature that emphasizes the conscious re-experiencing of past events. Current evidence suggests that modulations of BSC impact later memory, including objective EM performance and subjective autonoetic recollection (and other forms of memory such as working memory). These BSC–EM effects are supported by brain mechanisms in the posterior parietal cortex (particularly the temporoparietal junction and precuneus), motor cortex (premotor cortex and supplementary motor area) and prefrontal cortex that are coupled with the hippocampal complex, mediating BSC effects in EM. Linking subjective bodily experience during encoding and retrieval to the subjective re-experiencing of past events, we highlight open questions for future research on self and memory.

This article is part of the discussion meeting issue ‘From being to identity: analysis and synthesis of the self’.

## 1. Introduction

Memory broadly refers to the cognitive processes that enable individuals to encode, store and retrieve information [1]. Episodic memory (EM) is the only memory system that allows the conscious recollection of personally experienced events in rich detail [2,3], and successful episodic retrieval has consistently been associated with the reactivation of brain regions that were also engaged during encoding (i.e. neural reinstatement [4]). Important research has focused on the reinstatement of *contextual* information, such as sensory, perceptual or emotional features of an event. For example, auditory brain regions are reactivated when recalling events involving auditory stimuli [5]. However, the role of bodily signals, which provide a *bodily context* for memory processes, has largely been overlooked.

The present review focuses on the *bodily context* in EM, because all life events are intrinsically linked to the subject or observer who experiences them from within their body. During encoding, the body receives rich multisensory and sensorimotor signals, and the integration of these bodily signals gives rise to a form of self-consciousness known as bodily self-consciousness (BSC; [6–11]). BSC is a lower-level form of self-consciousness, characterized by subjective experiences such as first-person perspective and the sense of agency (see §2 for further details) and it has also been argued to be relevant to key subjective components of EM.

In the present review, we first introduce key concepts and experimental approaches for manipulating BSC, followed by a brief overview of different components of EM (including autonoetic consciousness) and how they are assessed. We then highlight recent empirical work demonstrating the relevance of BSC to these components of EM in healthy human participants. Finally, we conclude by discussing open questions for future research on the relationship between self and memory.

## 2. Bodily self-consciousness

BSC comprises three key components: self-identification (also called body ownership; the sense that one's body belongs to oneself [12]), self-location (the experience of being located within one's physical body, encompassing a specific volume in space [13,14]) and first-person perspective (1PP; egocentric or field viewpoint experienced as directed at the world and centred on one's body [15]). In addition to these three features, the sense of agency (SoA)—the feeling of initiating and controlling one's own bodily actions and their effects [16]—is a fourth core feature of BSC and is increasingly being studied as part of the *bodily context* in EM research.

BSC is malleable, and researchers have developed a variety of experimental paradigms to manipulate it in healthy individuals. These paradigms typically involve exposing participants to multisensory conflicts, i.e. spatiotemporal mismatches across bodily signals, such as visual, tactile, vestibular, proprioceptive, interoceptive and motor signals. Such manipulations can selectively and temporarily alter specific components of BSC. Below, we summarize common methods for manipulating BSC (see table 1 for a classification according to the primary targeted component, noting that each manipulation may also influence other components). Owing to space constraints, we do not discuss the neural mechanisms underlying BSC, which have been thoroughly reviewed elsewhere [8,32,33], including mechanisms related to SoA [16,34].

**Table 1.** Methods for manipulating BSC and the targeted components.

manipulation method	primary targeted component	additional influenced components
visual–tactile conflict (e.g. full body illusion [9,17])	self-identification	self-location, first-person perspective
presence vs. absence of virtual body [18,19]	self-identification	self-location
visual–interoceptive conflict (e.g. visual–cardiac synchrony [20,21], visual–respiratory synchrony [22])	self-identification	self-location, sense of agency
visual–vestibular conflict (e.g. out-of-body illusion [23,24])	first-person perspective, self-location	self-identification
perspective shifts [25,26]	first-person perspective	self-identification, self-location
visual-motor conflict [27,28]	sense of agency over the body	self-identification
active action vs. passive observation [29]	sense of agency over action outcomes	/
free vs. forced action choice [30]	sense of agency over action outcomes	/
predicted vs. unpredicted action effects [31]	sense of agency over action outcomes	/

Self-identification (or body ownership) can be modulated through synchronous versus asynchronous visuo-tactile stimulation. A classic example of illusory limb ownership is the rubber hand illusion, where synchronous stroking of a visible rubber hand and the participant's hidden real hand induces the illusory feeling that the seen rubber hand is one's own hand [35,36]. The induction of illusory body ownership over the rubber hand has been quantified using a variety of methods, including subjective reports [36], proprioceptive drift [35], physiological responses [37] and nociceptive drift [38], compatible with a probabilistic framework for multisensory perception (i.e. Bayesian causal inference [39,40]).

However, BSC is not simply the sum of local representations of individual body parts; rather, it is experienced as a global and unitary sense of being in a single body [6]. The rubber hand illusion does not capture this global-unitary aspect of BSC [6]. To investigate whole-body self-identification rather than local self-attribution to isolated body parts, the full body illusion was introduced [9,17]. This illusion is typically induced by applying tactile stimulation to the participant's physical body while presenting corresponding visual stimulation of touch on a virtual or mannequin body. When the tactile stimulation applied to the real body and the visual stimulation presented on the virtual body are synchronous, participants experience illusory self-identification with the virtual body [26]. The effects of the full body illusion can be quantified by subjective reports, skin conductance responses to threats directed at the virtual body [9,37] or changes in skin temperature [41].

Beyond self-identification, a second feature of BSC, self-location, can also be modulated by synchronous visuo-tactile stimulation during the full body illusion. This is experienced as an illusory drift in self-location towards the virtual body, reflecting a global change in where the self is experienced in space. Such changes in self-location have been measured using gait responses [17] or mental imagery [15,42] (see also [43–45] for other measures). Using an adaptation of continuous flash suppression, it has further been shown that these BSC changes in self-identification and self-location occur independently of participants' awareness of visuo-tactile stimulation but depend on whether the stimulation is synchronous or asynchronous [46]. Although classical manipulations of self-identification and self-location were primarily linked to exteroceptive and proprioceptive cues, recent work has extended the full body illusion to interoceptive cues by exposing participants to cardio-visual or pneumo-visual stimulation of a virtual body [11]. For example, synchronous flashing of a virtual body with participants' heartbeat has been shown to induce illusory changes in self-identification and self-location similar to those observed with visuo-tactile stimulation [20,47]. Moreover, the full body illusion has been found to modulate interoceptive processing, as reflected by heartbeat-evoked potentials [21] and activity in the insular cortex [48] (see [49] for a review of interoceptive processes).

1PP—the conscious experience of being directed towards the external world—constitutes the third key feature of BSC. Although typically aligned with the physical body, the experienced direction of 1PP can be displaced during out-of-body experiences. Out-of-body experiences are strong disruptions of BSC that may occur spontaneously in healthy or clinical populations [50,51], and have been linked to deficits in multisensory integration [52] and altered vestibular input [53–55]. Several visual–vestibular manipulations have been shown to experimentally induce out-of-body-like states, including illusory displacements of 1PP [23,24], highlighting the vestibular contribution to BSC. Visual-vestibular and visual-gravity conflicts have also been integrated into the full body illusion paradigm [56,57], inducing illusory changes of 1PP. In these cases, participants reported an illusory downward-looking perspective (compatible with a third-person perspective; 3PP), despite physically maintaining an upward-looking 1PP while lying in a supine position [15]. These 1PP-to-3PP changes resemble spontaneous out-of-body experiences, during which individuals report a disembodied viewpoint accompanied by elevated self-location and sensations of floating [50,52,55].

1PP is of great relevance to EM research but is typically studied using different terminology. In memory research, a distinction is often made between *field* and *observer* perspectives. Field perspective refers to re-experiencing past events through one's own eyes, as if looking out from the original point of view, whereas observer perspective involves recalling an event from an external vantage point, as if watching oneself from the outside, similar to out-of-body experiences [24] or experimentally induced perspective shifts during the full body illusion [25,26]. These perspectives map closely onto concepts commonly used in BSC research: 1PP resembles the notion of field perspective, whereas 3PP resembles observer perspective. We discuss field versus observer perspective manipulation further in §4, as they are frequently applied in research on episodic autobiographical memory.

SoA is another core feature of BSC and has been conceptualized in two ways: (1) within the core bodily context, as the sense of control over one's own body and actions (i.e. the feeling that I, not someone else, am the agent of my bodily movements), and (2) more broadly, as the sense of control over the outcomes of those actions (i.e. the feeling that my actions, not those of others, cause changes in the environment or in my body) [16,58]. The SoA over the body can be manipulated in virtual reality through synchronous or asynchronous visuomotor feedback: when virtual hand movements match real hand movements in space and time, the SoA is preserved, whereas mismatches reduce it [27,28]. Notably, this manipulation also influences self-identification. The SoA over action outcomes is typically manipulated at three levels [59]: (a) motor execution, in which participants either actively perform an action to produce a sensory effect (high SoA) or passively perceive the same effect [29]; (b) action selection, in which participants either freely choose which action to perform (free choice; high SoA) or perform a predetermined action (forced choice; [30]); and (c) action–effect contingency, in which participants' actions either reliably produce predicted outcomes (high SoA) or do not reliably control outcomes [31].

## 3. Episodic memory

EM is a form of long-term declarative memory that supports the retrieval of personally experienced events, including their sensory features and their unique spatial and temporal context [2,3]. It differs from other types of memory such as semantic memory, which stores factual knowledge [60], and working or short-term memory, which maintains information over brief intervals of a few seconds or minutes [61].

In laboratory settings, EM is commonly assessed using relational recognition tests (also called associative, source or context recognition tests), in which participants are asked not only to discriminate old from new items but also to retrieve contextual details such as where or when the item appeared. A fundamental distinction within episodic retrieval is between recollection and familiarity. Recollection involves the retrieval of specific contextual details from the original event, whereas familiarity refers to a sense of prior encounter in the absence of contextual information. Paradigms such as the remember/know task are routinely employed to dissociate these processes, with ‘remember’ responses indexing recollection and ‘know’ responses indexing familiarity.

Recollection is closely tied to autonoetic consciousness, a defining feature of EM that enables individuals to mentally project themselves into the past and re-experience events [62–65]. Autonoetic consciousness is typically measured using subjective ratings across experiential dimensions such as vividness, sensory detail, emotional intensity and visual perspective. In contrast to objective measures of memory strength (e.g. accuracy or reaction times in recognition tasks), autonoetic measures capture the qualitative characteristics of remembering and rely on participants' introspective evaluations of their recollective experience.

Beyond laboratory-based stimuli, episodic autobiographical memory refers to the retrieval of personally meaningful episodes embedded within one's life history [2,3]. These memories integrate specific episodic details with broader self-related and semantic knowledge. A key methodological challenge is the limited experimental control over the encoded events: there is typically no objective record against which to assess the accuracy of autobiographical retrieval. Consequently, episodic autobiographical memory is commonly assessed using cued-recall paradigms (e.g. recalling a specific life event related to ‘beach’) and through subjective ratings collected via questionnaires or structured interviews [66].

Although these constructs are conceptually related and partially overlapping, they highlight complementary facets of episodic remembering: episodic retrieval supported by familiarity and recollection processes, the subjective re-experiencing of past events (autonoetic consciousness) and the integration of specific episodes into a broader life narrative (episodic autobiographical memory).

## 4. Bodily self-consciousness in episodic memory

Here, we review paradigms and the effects of BSC manipulations on EM, addressing both behavioural outcomes and underlying neural mechanisms. We also briefly summarize the influence of BSC on other types of memory, such as working memory. Tasks are considered to assess EM when they require the recall or recognition of unique events enriched with spatial, temporal, perceptual, emotional or self-related details, and when encoding and retrieval are separated by at least one hour to allow for consolidation. The experimental designs of the reviewed studies and the associated brain regions are summarized in figures 1 and 2, respectively.

**Figure 1.**
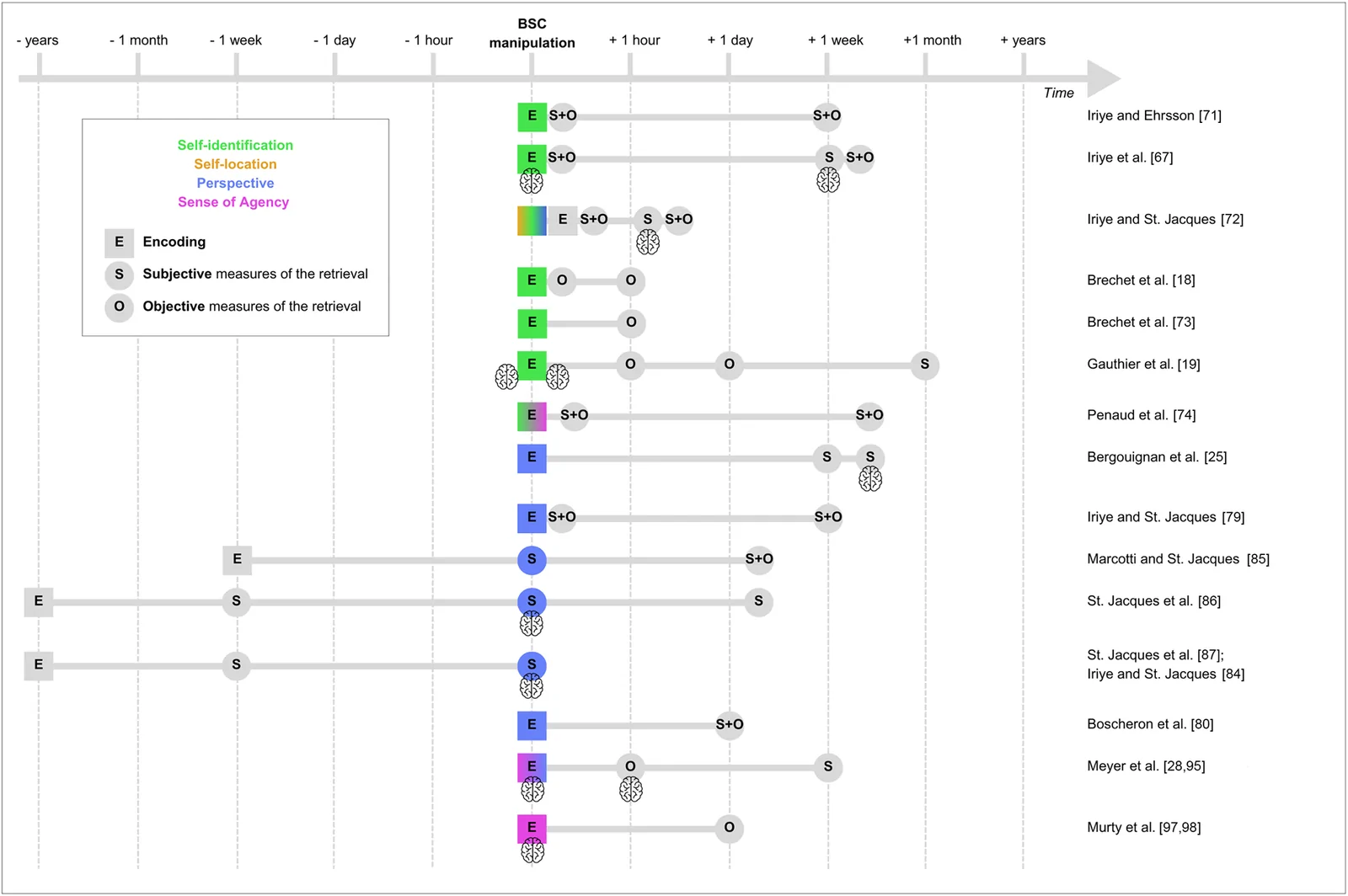
Overview of BSC manipulations in episodic memory research. For each study highlighted in this review, the figure shows the delay between encoding and retrieval (ranging from hours to years), the timing of fMRI acquisition (pre-encoding, post-encoding, during encoding, during retrieval or both), the memory phase at which BSC was manipulated (pre-encoding, encoding or retrieval) and the primary BSC component targeted. Retrieval was assessed using objective measures (e.g. recognition accuracy) and/or subjective measures indexing autonoetic consciousness (e.g. vividness ratings).

**Figure 2.**
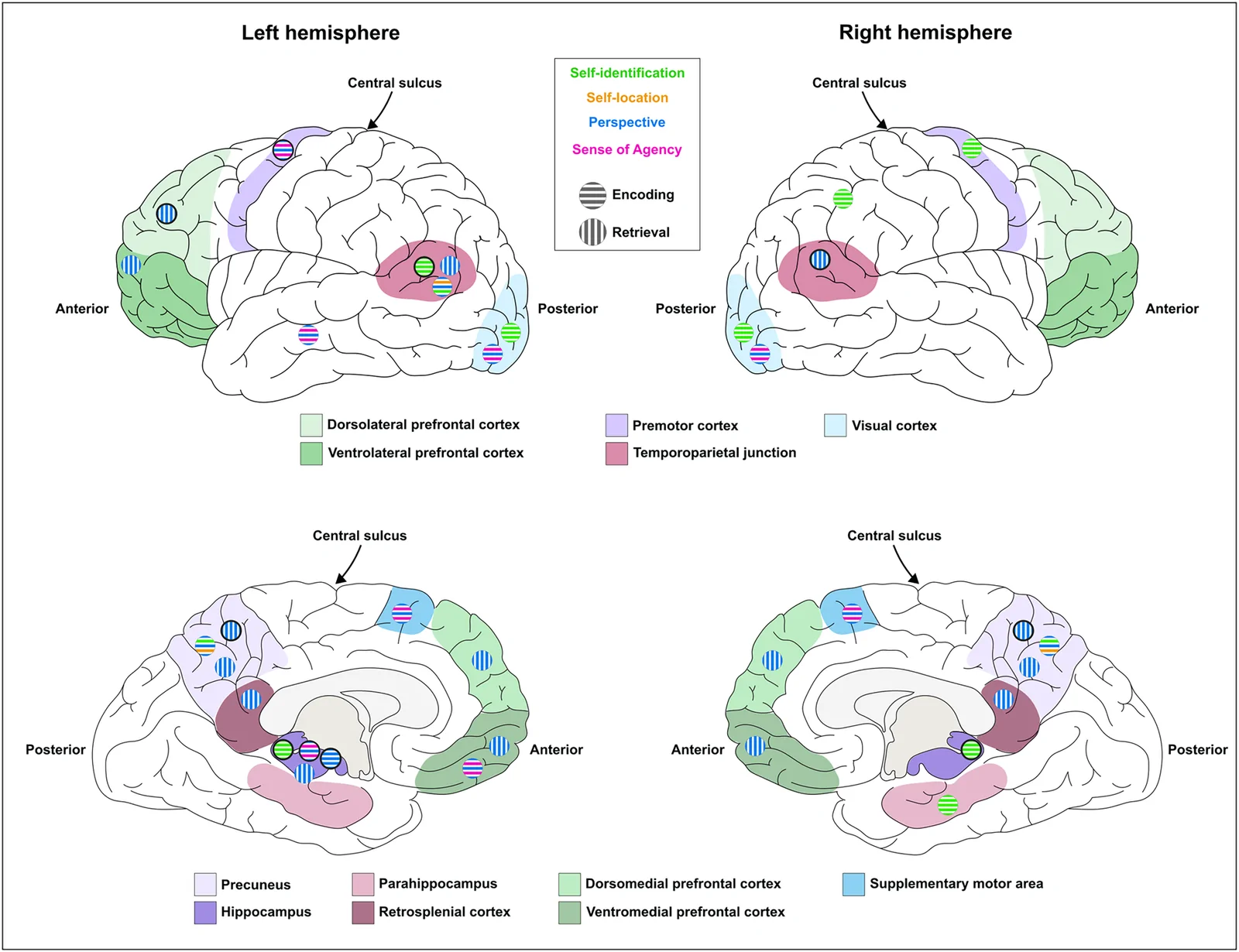
Brain regions showing BSC effects on episodic memory. The figure presents lateral (upper row) and medial (lower row) views of both hemispheres. Circles serve as markers placed on brain regions reported as active during manipulations of one or multiple BSC components in episodic memory paradigms, with circle colours denoting the manipulated BSC component. Horizontal lines within circles indicate manipulations applied during encoding, whereas vertical lines denote manipulations during retrieval. Circles with bold outlines mark regions that were not only activated when contrasting BSC conditions but also correlated with episodic memory measures (e.g. recognition accuracy, ratings of autonoetic consciousness). Regions of interest may be left uncoloured or highlighted in distinct colours (see legend below the figure for colour–region correspondence). Coloured regions of interest correspond to regions reported across multiple experiments or considered key nodes of BSC–EM circuits in previous literature. Additional empirical findings also reported activity in other regions (e.g. middle temporal gyrus, intraparietal sulcus), which are indicated by circles placed on uncoloured regions. Two activations—lateral cerebellum (reported in a single study [67]) and putamen (not visible from these views)—are not shown. The medial views are schematic representations combining multiple regions visible in near mid-sagittal slices; therefore, not all highlighted medial regions necessarily co-occur in a single anatomical plane. Regions identified solely through functional connectivity analyses are not included (see [29,68–70]).

### (a) Impact of self-identification/body ownership on episodic memory

Using visuo-tactile stimulation, Iriye and Ehrsson [71] manipulated participants' self-identification over a mannequin's body while they watched pre-recorded everyday events through virtual reality goggles. Surprise memory tests were administered immediately after encoding and again one week later (cued recall questions and subjective ratings for each event). The cued recall questions targeted either central (information essential to the main storyline) or peripheral (non-essential information to the main storyline) details. Subjective ratings assessed reliving, vividness, emotional intensity and perceived memory accuracy. The synchronous condition, which induced stronger self‑identification with the mannequin (as reflected in ratings), yielded greater recall accuracy for both central and peripheral details at both recall times. Regarding subjective ratings of autonoetic consciousness, the synchronous condition increased reliving scores immediately after encoding and enhanced emotional intensity, vividness and perceived memory accuracy one week later.

Using the same paradigm with functional magnetic resonance imaging (fMRI), participants were scanned during encoding and one week later during retrieval [67]. Whole-brain analyses revealed that activation during encoding in BSC regions (premotor cortex, intraparietal sulcus, temporoparietal junction), the lateral occipital cortex, putamen and cerebellum, as well as in memory regions (right hippocampus and parahippocampal gyrus), discriminated between strong and weak self-identification conditions. Additional region-of-interest analysis showed that the activation in posterior hippocampus during encoding also discriminated between the two conditions. To investigate the neural activations across memory phases, the authors computed the encoding–retrieval similarity, an analysis that quantifies how similar (i.e. high in correlation) the neural patterns of the same regions are between encoding and retrieval. The encoding–retrieval similarity, computed within the nine aforementioned regions, was higher for events encoded with strong self-identification (but only when trial-by-trial vividness ratings were high). This vividness-dependent effect was observed in the bilateral hippocampus and temporoparietal junction (no region showed differences in encoding–retrieval similarity between conditions independent of vividness). However, by contrast to the behavioural study, in this fMRI sample, memory accuracy and subjective ratings of autonoetic consciousness did not differ between conditions.

Another study manipulated not only self-identification (or body ownership) but also self-location and perspective [72]: Participants viewed their body from either 1PP or 3PP while receiving synchronous or asynchronous visuo-tactile stimulation in virtual reality. Immediately after each manipulation, they played four short virtual reality games with the experimenter to generate naturalistic and distinctive memories. After a 1.5–3-hour delay, participants retrieved each game and then rated vividness and perspective in the scanner. Additionally, immediately after encoding and again after the later scanning, participants answered cued recall questions and provided ratings of autonoetic consciousness. Memory accuracy in the post-scanning test was higher for the 1PP-synchronous condition than the 3PP-synchronous condition. However, no differences were observed between conditions in ratings of autonoetic consciousness (including vividness, emotional intensity and perceived memory accuracy). Region-of-interest analyses of the temporoparietal junction, precuneus and hippocampus revealed that activation in the left temporoparietal junction and bilateral precuneus distinguished BSC (self-identification and self-location), independent of perspective (perspective alone could not be discriminated based on activities in these regions). The left temporoparietal junction differentiated between 1PP and 3PP across both strong and weak BSC conditions, whereas the precuneus discriminated perspective only for events encoded with strong BSC.

Beyond manipulations of self-identification, research from our group has examined how the visibility of one's virtual body in virtual reality influences EM. In behavioural experiments, Bréchet *et al.* [18] found that participants remembered virtual scenes better when a virtual body was visible in a first-person view during encoding compared with conditions in which no body or a non-bodily control object was shown. This benefit emerged in a recognition test conducted one hour after encoding, but not immediately afterwards. In a follow-up study, the one-hour body-related memory advantage was replicated and further accompanied by a retrospective effect: when new scenes were encoded with the body present, memory was also enhanced for conceptually related scenes from an earlier body-absent phase [73]. This carry-over effect did not occur when both encoding phases lacked a visible body, suggesting a body-related tagging mechanism that enhances memory consolidation. Extending this line of work to neuroimaging, Gauthier *et al.* [19] recorded resting-state fMRI immediately before and after encoding, where participants viewed scenes either with or without a visible virtual body in the virtual reality task. Recognition memory was tested after one hour and after one day, and ratings of autonoetic consciousness were collected one month later. Although body visibility did not affect recognition accuracy or ratings of autonoetic consciousness in this study, region-of-interest analyses revealed stronger post-encoding functional connectivity between the right hippocampus and right parahippocampus in the body-visible condition. Moreover, post-encoding connectivity between medial temporal regions and primary somatosensory and motor cortices positively correlated with ratings of autonoetic consciousness, again only for scenes encoded with a visible body. This result is compatible with contributions of sensorimotor BSC mechanisms to EM.

Penaud *et al.* [74] manipulated both the visibility of the virtual body and the synchrony of visuomotor feedback. Participants navigated a virtual city under three conditions: (i) a synchronous avatar whose movements matched their own, (ii) an asynchronous avatar with delayed visuomotor feedback or (iii) no avatar (No-body). After encoding, they completed surprise free recall and recognition tests, administered both about 20 minutes later and again about 10 days later. During free recall, participants reported events, their egocentric spatial (in the left, right, or front of them) and temporal (at the beginning, middle or end of the district) context and event details. Recognition included old/new judgments, source memory and ratings of re-experiencing, vividness, confidence, emotional intensity and ownership. Asynchronous and No-body conditions reduced self-identification, self-location and SoA relative to synchronous feedback. Moreover, the asynchronous condition impaired the number of events freely recalled, perceptual details, contextual associations, subjective remembering and long-term consolidation. By contrast, the No-body condition mainly impaired egocentric spatial memory and reduced remembering only at the ten-day delay, supporting the belief that BSC changes during encoding modulate objective and subjective components of EM.

In addition to EM, BSC manipulations also affect other forms of memory. Tacikowski *et al.* [75] showed that embodying a friend's body through synchronous visuo-tactile stimulation (body-swap illusion) shifted self-trait ratings toward friend-trait ratings, depending on illusion strength. An immediate surprise recognition test revealed poorer memory for trait words encoded during the body-swap illusion, and reduced ownership of one's own body was associated with poorer memory. These findings suggest that self-identification (or body ownership) during encoding modulates memory for self-related words. Self-identification also influences spatial memory. Moon *et al.* [76] found that the visibility of an avatar from 1PP during virtual navigation improved spatial memory precision when tested immediately after encoding. Entorhinal grid-like activity as well as retrosplenial and intraparietal activity were modulated in the body-visible condition, with retrosplenial activity predicting memory precision. In another spatial memory study on grid-cell representation in the entorhinal cortex, it was shown that subjective changes in self-location (and self-identification), as induced by visual–tactile stimulation in the full body illusion, are sufficient to activate entorhinal grid-like activity [77], supporting that the bodily context and BSC level during encoding impact spatial memory and EM.

In summary, several BSC–EM studies have manipulated self-identification (or body ownership) and the visibility of the participant's body in virtual reality, either during or immediately before memory encoding. Most studies report that elevated self-identification and visibility of a virtual body strengthen objective memory measures (e.g. recognition or cued recall accuracy), whereas findings on subjective measures (i.e. ratings of autonoetic consciousness) are mixed. In fMRI studies, investigations have mainly focused on predefined BSC- and memory-related regions using region-based approaches, although whole-brain analyses have also been conducted. Activation findings show that key brain regions mediating BSC–EM effects include the temporoparietal junction and the lateral and medial posterior parietal cortex, as well as hippocampus and parahippocampus. Post-encoding resting-state connectivity analysis further reveals altered hippocampus–parahippocampus coupling, highlighting the pivotal role of the hippocampal complex in BSC–EM mechanisms related to self-identification. In our studies on spatial memory and navigation, activations in entorhinal and retrosplenial regions have also been shown to be modulated when self-location is manipulated in addition to self-identification.

### (b) Impact of perspective on episodic memory

Several studies have experimentally manipulated participants' perspective (1PP versus 3PP) *during encoding* to explore its influence on EM. Bergouignan *et al.* [25] induced a full-body illusion in virtual reality using visuo-tactile stimulation while participants engaged in a scripted real-life social interaction. In the in-body condition, the event was experienced from 1PP, whereas in the out-of-body condition, participants felt displaced outside their physical body and viewed the interaction from a position and perspective that was a few metres away (i.e. changes in self-location and the direction of the 1PP), comparable to so-called out-of-body experiences [15,52,78]. Memory was assessed one week later with a structured interview (Experiments 1 and 2), in which participants freely recalled the episode, and responses were scored based on the number of episodic details retrieved (including emotion, what, where and when). Recall of episodic details was significantly reduced for out-of-body-encoded events, suggesting that shifted perspective and distanced self-location at encoding disrupt autonoetic consciousness during retrieval. In a follow-up fMRI experiment (Experiment 3), memory retrieval was tested in the scanner 10–14 days after encoding. Participants were cued to repeatedly recall each event and rate its vividness, retrieval difficulty, emotional salience and adopted perspective. Whole-brain analyses showed reduced activation for out-of-body-encoded events compared with in-body-encoded events in the left posterior hippocampus. Region-of-interest time-course analyses of the left posterior hippocampus further revealed distinct activation patterns between conditions: for in-body memories, hippocampal activation was strong for the first retrieval trial and decreased with repetition, whereas for out-of-body memories, hippocampal activation was initially weak and increased progressively across repetitions. Moreover, reduced hippocampal activation was correlated with reduced vividness of out-of-body memories. These findings suggest that preserved BSC during encoding facilitates rapid, vivid memory retrieval, whereas out-of-body state during encoding hampers hippocampal retrieval processes, leading to weaker and less vivid memories. In another study, Iriye and St. Jacques [79] manipulated perspective alone during encoding in a virtual reality task, but they could not observe differences in objective memory accuracy (cued-recall test for visual details; map-drawing task for spatial layout). Likewise, there were no differences in ratings of autonoetic consciousness, including perspective, vividness, reliving and emotional intensity of the memories after a one-week delay, suggesting that further work is needed using perspective-related BSC manipulations during encoding.

In addition to shifts between 1PP and 3PP, a recent study from our group manipulated participants' viewpoint toward objects (i.e. participant–object angle) within 1PP during lifelike interactions with objects in a virtual reality task. One day later, participants completed a surprise memory test in which they first recalled the event associated with the object and rated their autonoetic consciousness regarding the extent to which they re-experienced the memory and the visual vividness of the memory. They were then re-immersed in the virtual environment and asked to retrieve their original viewpoint relative to the object. Results showed that viewpoint retrieval accuracy varied as a function of the encoding angle and, critically, was correlated with vividness ratings. These findings demonstrate a direct impact of the 1PP encoding angle on both autonoetic consciousness and episodic recall of viewpoint [80].

Several studies have examined how adopting different perspectives *during retrieval* influences EM—particularly episodic autobiographical memory. Converging evidence shows that recalling events from 1PP or a field perspective is typically associated with higher ratings of autonoetic consciousness, including vividness, emotional intensity, personal importance and sensory details compared with recalling from 3PP or an observer perspective [81–86]. Additionally, Marcotti and St. Jacques [85] found that intentionally recalling lab-generated events from 3PP versus 1PP one week after encoding not only reduced vividness at retrieval but also decreased accuracy in a cued-recall test performed two more days later. Overall, these findings indicate that active perspective shifting during retrieval can alter the underlying memory representation, with lasting consequences for the fidelity of later recall. Other studies investigated the brain mechanisms underlying the effect of perspective changes on episodic autobiographical memory. St. Jacques *et al.* [86] asked participants to generate hundreds of autobiographical memories from the past 5 years. The memories most strongly tied to field/1PP were then selected for a cued retrieval session one week later in the MRI scanner. During scanning, participants were asked to recall each memory either from its original field/1PP (no perspective shift) or from the view of an observer/3PP (perspective shift) (each memory was retrieved three times to permit repetition suppression analyses; participants rated emotional intensity and ability to recall from the instructed perspective). Two days later, all memories were freely recalled without perspective instructions, followed by ratings of perspective, reliving and emotional intensity. Behaviourally, observer/3PP recall was rated as more difficult and led to slower responses and lower emotional intensity compared with field/1PP recall. Moreover, memories intentionally shifted into observer/3PP were more likely to be spontaneously recalled from that perspective in the later session. Whole-brain activation analyses showed repetition suppression in both conditions in classical episodic autobiographical memory networks, including the medial temporal lobe, ventromedial and dorsomedial prefrontal cortices, lateral temporal cortices, inferior posterior parietal cortices, posterior midline and the cerebellum. However, stronger suppression emerged in the precuneus and right angular gyrus during observer/3PP recall, probably indicating their role in perspective taking during retrieval. Trial-level analyses further revealed that the amount of repetition suppression in the precuneus predicted both reductions in emotional intensity during observer/3PP retrieval and long-term perspective shifts across sessions. By contrast, left prefrontal cortex activity was more strongly associated with maintaining the original field/1PP, pointing to distinct perspectival mechanisms depending on retrieval demands.

In two follow-up studies, St. Jacques *et al.* [87] further showed that, within the retrieval-specific regions identified using whole-brain activation analyses, shifting perspective during the retrieval of episodic autobiographical memory engaged the ventromedial, posterior dorsomedial, ventrolateral and dorsolateral prefrontal cortex, as well as the temporoparietal junction and medial posterior parietal cortex (including the posterior cingulate cortex). Region-of-interest analyses further revealed that the precuneus was sensitive to perspective changes, regardless of whether memories were recalled from 1PP or 3PP. Additionally, using a multivariate analysis approach, Iriye and St. Jacques [84] found that field/1PP retrieval elicited stronger activation across the typical network of episodic autobiographical memory compared with observer/3PP. Functional connectivity analyses using an anterior hippocampal seed further revealed different spatiotemporal dynamics in the network of episodic autobiographical memory across perspectives. Specifically, initial 3PP retrieval activated stronger connectivity between the hippocampus and different posterior parietal regions (precuneus, angular gyrus, retrosplenial cortex) and posterior parahippocampal cortices, whereas later stages of 1PP retrieval involved stronger connectivity within the medial temporal lobe and between the hippocampus and ventromedial prefrontal cortex.

In summary, perspective manipulations during encoding typically involve the creation of lab-based, life-like events and are often coupled with changes in self-location. Such manipulations have been shown to influence both objective memory performance and subjective experiences of autonoetic consciousness, although not consistently across studies. One fMRI study using whole-brain activation analyses implicated the hippocampus in these effects. Studies of 1PP/3PP effects during retrieval have mostly relied on real-life autobiographical memories, for which objective accuracy cannot be measured, and have thus focused on subjective experience. Region-of-interest analyses within retrieval-specific regions showed that 3PP during recall was associated with the medial posterior parietal cortex (precuneus, retrosplenial cortex) and temporoparietal junction, and these areas are all key regions supporting perspective taking [68,88–90] as well as egocentric–allocentric transformations in spatial navigation and memory [91–94]. By contrast, the prefrontal cortex was implicated in 1PP during retrieval. However, further work is needed to corroborate these findings, establish clearer links to subjective and objective memory measures, and include controls for non-mnestic and non-BSC attentional and imagery-related aspects. There is currently little evidence for BSC-related hippocampal and parahippocampal contributions.

### (c) Impact of sense of agency on episodic memory

Our group has recently conducted a series of studies investigating whether, and how, the SoA over an avatar's body influences EM and autonoetic consciousness. Meyer *et al.* [28] induced different levels of the SoA during encoding by varying visuomotor synchrony (synchronous versus asynchronous) and perspective (1PP versus 3PP) while participants encoded virtual scenes in virtual reality. Participants experienced three conditions: 1PP with synchronized movements between the real and virtual hands, 1PP with asynchronous movements, and 3PP with asynchronous movements. Immediately after encoding, BSC in each condition was measured in a new virtual scene to avoid interference with encoding. SoA ratings were higher in the 1PP-synchronous condition compared with the other two conditions, with no robust differences observed between the 1PP-asynchronous and 3PP-asynchronous conditions. Body ownership ratings were also higher in the 1PP-synchronous condition compared with the 3PP-asynchronous, but not compared with the 1PP-asynchronous condition. Whole-brain univariate analysis comparing the 1PP-synchronous condition with the other two revealed activation of a SoA-related network in left dorsal premotor cortex and bilateral supplementary motor cortex, with activity in these regions correlating with SoA ratings but not with body ownership ratings. Recognition accuracy, tested one hour after intentional (not incidental) encoding, was enhanced in the 1PP-synchronous condition relative to the other two conditions, while no difference emerged between the 1PP-asynchronous and 3PP-asynchronous conditions. Whole-brain searchlight representational similarity analysis, measuring both the neural activation and the similarity of activations between encoding and retrieval, showed that the SoA manipulation modulated brain activity in the left hippocampus, visual cortex, left middle temporal gyrus and left frontal superior orbital gyrus. Among the four regions, hippocampal encoding–retrieval similarity was significantly higher in the 1PP-synchronous condition compared with the other two conditions, and this similarity predicted the behavioural recognition performance. Moreover, coupling between hippocampal encoding–retrieval similarity and premotor encoding–retrieval similarity (a SoA-related region) varied by condition, indicating that SoA at encoding was reinstated during EM retrieval. In this work, autonoetic consciousness was measured one week after encoding using questionnaires [95], and ratings were highest for events encoded in the 1PP-synchronous condition. Critically, only in this preserved-SoA condition (i.e. 1PP-synchronous), ratings of autonoetic consciousness were positively associated with SoA ratings (not body ownership) during encoding; and this behavioural SoA–autonoetic consciousness link was mediated by premotor–hippocampal coupling. Together, these findings link the subjective SoA during encoding to the later subjective re-experiencing (autonoetic consciousness) of these episodes.

In another experiment, Meyer *et al.* [96] recorded resting-state fMRI immediately before retrieval, which took place one week after encoding. They found that better memory performance per subject in the preserved-SoA condition was associated with stronger functional connectivity between the right hippocampus and the right superior parietal lobule, whereas better performance in the disrupted-SoA condition (i.e. 3PP-asynchronous) was linked to stronger connectivity between the right hippocampus and the left angular gyrus, indicating different neural mechanisms for the memory consolidation of events encoded under preserved and disrupted SoA. This series of experiments manipulated both SoA and perspective. The observed effects on EM are primarily interpreted as being driven by changes in SoA, given the associations among SoA ratings, behavioural memory performance and brain activity; however, the additional inclusion of a fourth condition (synchronous 3PP) may have allowed further disentanglement of the impact of perspective vs. sensorimotor congruency.

Broader evidence for SoA–EM coupling comes from studies manipulating SoA, not over an avatar's body, but over external action outcomes. When individuals freely controlled the presentation of visual items versus other conditions characterized by forced choice [69,97,98] or passive observation [70], their memory for those items and their spatial locations, tested one day or one week later, was often enhanced. Whole-brain analyses showed stronger activation in the left striatum and bilateral anterior temporal lobes during the presentation of the free-choice cue compared with the fixed-choice cue. More importantly, region-of-interest analyses showed that only in the free-choice condition, the left striatal activation (implicated in decision-making and motivation; [99]) predicted later memory performance [97]. In the free-choice condition, the single-trial cue-evoked striatal responses were also coupled with right hippocampal activation during successful encoding [97] and with increased post-encoding interactions between the hippocampus and perirhinal cortex [98]. Although these studies did not directly measure SoA, free-choice conditions are typically associated with stronger SoA [30,100]. Therefore, these studies suggest that SoA during encoding may facilitate EM through striatal–hippocampal interactions, complementing the premotor–hippocampal mechanisms implicated in effects of bodily SoA on EM.

Building on this line of research, our recent work directly examined whether SoA over action outcomes modulates EM [101]. During intentional encoding, participants pressed keys that caused a centrally presented object to move either congruently with their chosen direction or incongruently in the opposite direction. One hour later, they completed a memory test assessing item recognition (Old/New) and spatial memory (final object location). Participants reported higher SoA ratings in congruent than incongruent trials. Stronger SoA was associated with faster item-recognition responses, without changes in accuracy, suggesting enhanced decision efficiency or confidence rather than improved item-memory quality. By contrast, SoA ratings predicted higher spatial memory accuracy. Importantly, this SoA-driven spatial memory advantage is not explained by differences in encoding duration, spatial bias, or guessing strategies. Together, these findings suggest that experiencing control over actions during encoding selectively strengthens memory for the spatial consequences of one's actions, suggesting that SoA functions as a binding mechanism that links actions to their outcomes, prioritizing episodic memories of self-generated events.

Beyond memory for individual items, other studies show that SoA also benefits associative memory. Ruiz *et al.* [102] employed a game show-like paradigm where participants helped unique ‘contestants’ choose one of three doors. In the free-choice condition, participants freely selected a door, whereas in the forced-choice condition they had to select a previously highlighted door. After each choice, the prize behind the chosen door was revealed (we note that the ‘prize’ was simply a visual outcome of participants' choices and did not constitute a real monetary or point-based reward). Across experiments, free-choice consistently improved memory—not only for contestants but also for contestant–prize, contestant–door and door–prize associations. Additionally, the memory benefit for inferred door–prize associations emerged only when choices served an explicit goal. Again, although SoA was not directly measured, the authors proposed that SoA strengthens associative memory by facilitating inferential processes that bind overlapping elements across events. Finally, Houser *et al.* [103] found that active exploration of objects in an online game enhanced recall of event sequences compared with passive observation of objects, while memory for spatial locations and item features did not differ.

SoA over external action outcomes during encoding has also been tested on short-term memory. Hon and Yeo [104] showed that recognition of neutral words was enhanced when participants experienced stronger SoA over their movement on the screen. Words presented under high-SoA conditions (i.e. spatially congruent, 100 ms delay between keypress and word movement) yielded higher recognition hit rates in an immediate surprise test, compared with low-SoA conditions (i.e. incongruent or with a 900 ms delay). Using similar paradigms, two studies replicated such SoA-driven memory enhancements [105,106], yet one study failed to replicate the effect under both intentional and incidental encoding [107]. There is also evidence for SoA effects on working memory [108]. Participants moved several boxes on a screen using the arrow keys, one of which differed in controllability. Afterwards, an icon appeared inside each box for encoding, followed two seconds later by a recognition test for one of the icons and SoA ratings. Authors observed better memory for items associated with higher SoA, suggesting that SoA contributes to working memory.

In summary, most current evidence comes from studies manipulating SoA over action outcomes during encoding, while research on the bodily SoA and EM has only recently emerged. SoA manipulations during encoding have consistently been shown to facilitate EM, and a higher SoA facilitates not only item memory but also the binding of items to their associative contexts (e.g. temporal, spatial), mediated via striatal–hippocampal or premotor–hippocampal coupling. However, most past work did not directly measure SoA; when SoA was measured, ratings were obtained post-encoding rather than on a trial-by-trial basis. Neural data linking SoA to memory performance and autonoetic consciousness remain scarce.

## 5. Why does bodily self-consciousness contribute to episodic memory?

The studies reviewed here are heterogeneous in terms of the BSC components manipulated, the memory phases at which BSC was altered and the EM tasks and measures used. Despite this variability, the general takeaway is that EM is impaired when the bodily context is disturbed. Episodes encoded under impaired self-identification or decreased SoA tend to be remembered less accurately than those encoded with a non-disturbed body context. Findings from experiments manipulating perspective further suggest that the disturbed contribution of the bodily context to memory persists in time. Notably, all studies summarized here use tasks without explicit reward contingencies, indicating that the observed BSC effects on EM are unlikely to reflect reward-related processes, which are known to enhance memory [109].

Why does BSC contribute to EM? One compelling reason may be that the normal BSC state, comprising self-identification, self-location, 1PP and SoA, constitutes a ‘default’ bodily state for encoding and retrieving everyday events. This default state is grounded in lifelong multisensory–sensorimotor learning: over the course of development, individuals build predictive sensorimotor representations and construct internal forward models that shape the perception of the body and external world and link actions to their sensory consequences [110,111]. Although this learning process unfolds across massive physical changes of the body in one's lifetime (e.g. changes in body size, form and fitness from childhood to adulthood), the core components of BSC remain or seem to remain remarkably stable. This preserved BSC context provides a consistent subjective bodily state across time, such that retrieval usually occurs under conditions that match the BSC present during encoding, thereby facilitating contextual reinstatement. When BSC is experimentally altered at encoding or retrieval, such manipulations may violate long-established sensorimotor predictions and introduce a bodily context mismatch, thereby disrupting contextual reinstatement and reducing EM performance, revealing that BSC makes important contributions to EM.

This perspective is consistent with, but also extends, classic memory theories. Context-dependent memory theories [112] highlight that memory benefits when external contextual cues at retrieval match those present at encoding; for example, people recall more information when tested in the same physical environment in which they learned it. Sensorimotor models [113] expand this principle to the ‘internal’, objective bodily context: stable multisensory–sensorimotor patterns (e.g. body postures) serve as contextual cues that are reinstated during retrieval. Notably, we argue that the BSC context goes beyond this physical ‘bodily context’ in two critical ways. First, BSC adds a self-conscious layer to the bodily state or context. The body is not merely a constellation of sensorimotor signals; it is experienced as *my* body, from *my* perspective, and it executes *my* actions. This transforms bodily context from an objective physical background-context into an integral component of the subjective experience of any episode. Second, BSC provides the experiential bridge between the self at encoding and the self who later re-experiences the event. This continuity underpins autonoetic consciousness, the hallmark of EM: the awareness that I, now, am recalling and vividly re-experiencing what I lived at a particular moment in the past. Taken together, we propose that BSC influences EM not only through a bodily contextual reinstatement, as predicted by classic theories, but also by providing critical signals for the subjective experience of remembering, thereby turning past episodes into episodes of someone, of my past.

Concerning the BSC–EM brain mechanisms, the reviewed neuroimaging findings, though also heterogeneous, reveal several brain regions that are consistent across BSC–EM tasks. The hippocampus, a central structure in EM, is the region most frequently implicated in BSC–EM studies. It was found to differentiate between BSC conditions in terms of activation patterns (high vs. low levels of sense of ownership [67] and SoA [28]), temporal dynamics of neural activity [25] and functional connectivity (stronger post-encoding connectivity between the right hippocampus and right parahippocampus when a visual body is present in virtual reality [19], stronger connectivity between the right hippocampus and right superior parietal lobule with normal SoA levels, and stronger connectivity between the right hippocampus and left temporoparietal junction with disturbed SoA [96]). In addition, we observed that left hippocampal activity differentiates between different BSC states, whereas the right hippocampus displays different connectivity patterns with other regions. However, suchhippocampal differentiation is not observed consistently across studies (even when examined using a region-of-interest approach [72]) and given the sparsity of studies, this should be regarded with caution.

Our review also highlighted the role of the temporoparietal junction in BSC–EM coupling. The temporoparietal junction, a core sensorimotor BSC region, is engaged during episodic autobiographical memory retrieval [87]. It differentiates between multiple BSC components, including high vs. low sense of ownership [67], self-identification [72] and 1PP vs. 3PP [72], supporting a general role in coordinating BSC–EM interactions. Functional connectivity findings point to the importance of the angular gyrus, a subregion of the temporoparietal junction that shows significant connectivity with the hippocampus during experimentally modulated BSC states (3PP vs. 1PP [84], altered SoA levels [96]). Past data suggest that the temporoparietal junction may become particularly involved when the normal BSC state is experimentally modulated, helping to adjust or reinterpret the bodily context during retrieval. Again, given the limited number of studies, strong conclusions cannot be drawn. In comparison with the temporoparietal junction, the precuneus also differentiates between 1PP and 3PP, but only when other BSC components remain intact [72], suggesting a more specific role in linking EM and BSC. Regarding the remaining regions that appear in the cited literature, the frequency of reported neural activation or functional connectivity is very low (one or two studies). We acknowledge this scarcity of empirical evidence as a limitation that we will address in §6, among other issues to be targeted by future BSC–EM research.

## 6. Outlook and future directions

Despite growing interest in the influence of BSC on memory, especially EM, the number of studies in this area remains relatively low. Moreover, not all studies have directly investigated whether their method actually modulated BSC (i.e. they only tested multisensory stimulation on EM, but not on BSC in their participants), nor how such modulation varied across participants or single trials. Most studies have manipulated BSC during encoding (either self-identification/body ownership, self-location or SoA), while BSC manipulations during consolidation (to our knowldge, no studies to date) or retrieval (primarily perspective manipulations) have received much less attention. A further challenge is that multiple BSC components are often manipulated simultaneously, making it difficult to determine whether specific components contribute uniquely to EM or whether some exert stronger effects than others. Future research should therefore aim to manipulate BSC components independently to identify potential component-specific effects and directly compare how different components influence EM.

The reviewed neuroimaging evidence is also concentrated on encoding. Future studies should extend BSC manipulations and brain activity acquisitions across encoding, consolidation and retrieval to capture the full temporal evolution of how the brain associates BSC and memory, determine whether BSC effects are transient, contribute to consolidation, or persist over extended delays, and examine how these effects unfold across multiple time points. Additionally, although SoA is typically discussed in relation to externally directed actions [16], such as using one's hand to lift a water bottle, it also encompasses control over internal bodily states [58,114], such as intentionally modulating one's breathing to slow the heartbeat; however, to our knowledge, no study has yet examined how SoA over internal bodily states influences EM. Likewise, we are not aware of any study that has examined how manipulating interoceptive signals (e.g. cardiac signals) influences EM, despite their established role in shaping BSC [11,58], memory of words [115] and other cognitive and emotional systems [116,117].

Another underexplored factor is the role of encoding intentionality; that is, whether participants are informed before encoding that a memory test will follow and thus intentionally attempt to remember the events. Existing studies differ in whether memories are encoded intentionally or incidentally, and this factor may interact with BSC in shaping memory outcomes. For example, SoA-related memory advantage has been observed when encoding was intentional but not when it was incidental, with further interactions involving attention [28]. Future research should directly compare intentional and incidental encoding conditions to clarify how BSC modulates memory under different goal states, including how SoA-related effects depend on processes of action selection and outcome [59,118]. In addition, studies reporting effects of SoA on working or episodic memory typically manipulate SoA by varying sensorimotor congruency. This makes it difficult to determine whether SoA itself contributes to memory, or whether subjective SoA simply serves as a proxy for sensorimotor congruency mechanisms that drive the memory effects. Future work could attempt to partially dissociate subjective SoA from objective sensorimotor congruency, for example, by manipulating participants' beliefs about control while keeping sensorimotor congruency constant, or by using stable perturbations to which participants gradually adapt. Trial-wise modelling that includes both objective congruency and subjective SoA ratings, or mediation analyses testing whether congruency influences memory via SoA, would help to clarify whether SoA accounts for additional variance in memory performance beyond that explained by sensorimotor congruency.

Addressing these different methodological and conceptual gaps will advance the field towards a more comprehensive, mechanistic understanding of how BSC shapes remembering and re-experiencing events from one's past in terms of behavioural mechanisms and will also advance current neurobiological memory models [119,120].

Regarding brain imaging analysis, two strategies are commonly used in the studies summarized above. One strategy involves first conducting whole-brain analyses to identify regions involved in memory encoding or retrieval (e.g. comparing brain activity for events later remembered vs. forgotten), followed by region-of-interest analyses within these regions to determine which are sensitive to BSC manipulations. Another strategy starts by using whole-brain analyses to identify regions responsive to BSC manipulations and then applies region-of-interest analyses to examine coupling between these BSC-related regions and classical memory regions such as the hippocampus. Overall, whole-brain analyses should be favoured over predefined region-of-interest approaches, as whole-brain analyses more robustly reveal the networks supporting BSC–EM coupling. However, we also note that different studies have focused on different analytic approaches, including univariate and multivariate activity measures, functional connectivity, representational similarity between encoding and retrieval and brain–behavior coupling, and that not all studies use the same set of methods. This heterogeneity makes direct comparisons of neuroimaging findings across studies challenging. Also, most existing neural evidence comes from fMRI, which offers high spatial but limited temporal resolution. Complementary methods such as EEG/MEG and intracranial recordings are therefore indicated to track the dynamics of BSC–EM interplay. Potential confounding factors—such as visual field size (particularly when shifting perspective), encoding duration, emotional salience, attentional and mental imagery engagement, and motor activity (particularly when manipulating SoA)—should be carefully controlled to ensure that observed effects can be attributed to BSC and not to other factors.

Concerning virtual reality methods, many recent studies on BSC and EM have increasingly adopted virtual reality technology. For BSC manipulations, virtual reality allows flexible control over the synchrony between visual, auditory and sensorimotor contingencies, as well as shifts in self-location and perspective that are difficult to achieve with traditional methods. For EM, virtual reality also enables the simulation of rich scenarios that closely resemble real-life experiences while preserving precise experimental control. Future work should employ a larger variety of social scenarios optimized for memory research and further improve immersiveness, e.g. by enhancing the fidelity of avatars, stimuli and environments, which in some paradigms remain overly simplified or cartoon-like. Additionally, existing virtual reality tasks generally rely on custom-made stimuli. Developing large, shared stimulus sets (e.g. 3D objects) with normative ratings of familiarity, complexity and emotional valence (similar to established picture databases in traditional memory research) would greatly facilitate consistency and replication in virtual reality-based studies. Although two normative datasets comprising 124 and 147 realistic 3D objects are already available [121,122], ongoing advances in virtual reality technology and increasing experimental demands require the continuous refinement and expansion of such resources, including higher graphical fidelity and a broader range of objects to support diverse research paradigms.

Finally, we believe that detailed clinical studies would be important to further understand the BSC–EM brain mechanisms. We recently tested a patient with severe amnesia (including episodic autobiographical memory) but preserved global cognitive function, caused by bilateral hippocampal atrophy (infectious origin). While this patient was normally sensitive to BSC manipulations, the BSC effects on EM differed from those of healthy controls [28,96]. In fact, EM performance showed the opposite pattern: whereas healthy participants usually benefit from preserved normal BSC during encoding, the patient consistently exhibited detrimental effects. Specifically, preserved-BSC encoding led to poorer recognition accuracy, greater difficulty retrieving the viewpoint adopted at encoding and a reduced ability to re-experience the episodes (measured by ratings of autonoetic consciousness), suggesting that hippocampal damage and/or hippocampal disconnection from premotor, medial posterior parietal, or the temporoparietal junction impairs normal BSC–EM coupling. Beyond patients with memory disorders such as Alzheimer's disease and other dementias, individuals with deficits in sensorimotor integration—such as those with altered brain networks including the hippocampus or premotor cortex—may exhibit reduced coupling between BSC and autonoetic consciousness. For example, schizophrenia has been associated with disturbances in SoA [123,124], alongside reduced autobiographical memory [125,126]. Likewise, stroke patients with lesions in the premotor cortex or some of the other BSC–EM regions may also suffer from disrupted BSC–EM coupling. Even transient states such as paresthesia, partial anesthesia of a body part, or pain, which are known to reduce SoA and/or body ownership [127–131], may impact memory processes. Finally, BSC–EM research may also be warranted in dissociative disorders and post-traumatic stress disorders [132,133].

In conclusion, BSC provides a fundamental, yet underexplored, dimension of memory research. Theoretically, life events are not only defined by external and contextual features such as what, where, and when, but are also intrinsically tied to the “who” or sense of self. Here, we grouped evidence suggesting that multisensory bodily stimuli, as well as interoceptive signals and motor processes, are encoded along with other contextual information during an event and reinstated during retrieval. These same signals are also of fundamental importance for BSC and may thus provide an important bodily context for EM. Evidence reviewed here supports the view that BSC manipulations in healthy participants can modulate EM and other forms of memory. That bodily cues and the associated BSC provide an embodied bodily context when encoding and recalling life events is a physical reality that has only received scant attention from researchers. More BSC–EM research is needed to investigate the involved brain mechanisms, advancing memory research but also research on the sense of self. As Tulving famously stated, ‘No traveler, no travelling,’ emphasizing that the ‘traveler’, or the self, is central to episodic recollection [2]. Traditionally, this self was framed in terms of higher-order selfhood (cognitive or remembered self; [134–136]), which involves reflective processes that enable individuals to self-attribute not a body, perspective, or action, but to experience a thought or a perception as belonging to the self and as self-generated. Memory and cognitive self are deeply intertwined. First, the cognitive self contributes to episodic remembering, as it entails awareness of oneself both in the past (at encoding) and in the present (at retrieval) when re-living personal experiences. Second, episodic memories contribute to constructing and sustaining the cognitive self, weaving life events into a coherent personal narrative that underpins continuity and identity across time. Third, the evidence summarized here extends this by showing that BSC also contributes to EM, particularly to the re-experiencing of life events, autonoetic consciousness. The work by Donna Addis and colleagues described important facets of EM, such as diachronic unity that may have critical BSC components as well as semantic components [64]. Diachronic unity is the belief that, despite the many physical changes, we are the same person across our lifespan. Diachronic unity is based on phenomenological continuity (re-experiencing critical life episodes over time during episodic recall) and semantic continuity (the ability to construct life narratives from semantic memory), and thus on episodic and semantic components. We argue that global–unitary aspects of BSC (such as self-identification with a body and 1PP) may contribute to both temporal and unitary aspects of the self in time, especially phenomenological continuity. We hope that future research will integrate BSC models into models of memory to provide a more comprehensive and mechanistic account of how the lower-level bodily and higher-level cognitive dimensions of self jointly support the richness, continuity and identity of personal experience.

## Data Availability

This article has no additional data.

## Theme

One contribution of 17 to a discussion meeting issue ‘From being to identity: analysis and synthesis of the self’.
